# Preclinical Safety and Immunogenicity Characterization of Emulsivant 70: A Promising Candidate Adjuvant for Emulsified Veterinary Vaccines

**DOI:** 10.1155/jimr/7245322

**Published:** 2026-11-06

**Authors:** Caroline Dohanik, Leandro Gonzaga de Oliveira, Roberta Ondei, Dennis Christensen, Diego dos Santos Ferreira, Lucas Antônio Miranda Ferreira, Ricardo Tostes Gazzinelli, Ana Paula Fernandes, Gisele Assis Castro Goulart

**Affiliations:** ^1^ Department of Pharmaceutical Products Faculty of Pharmacy Universidade Federal de Minas Gerais Belo Horizonte 31270-901 Minas Gerais Brazil ufmg.br; ^2^ Detechta BH-TEC Belo Horizonte 31310-260 Minas Gerais Brazil; ^3^ Croda do Brasil Rua Croda 580 Campinas 13054-710 São Paulo Brazil; ^4^ Croda Pharma Vaccine Adjuvant Systems R&D Diplomvej 381 Lyngby DK-2800 Denmark; ^5^ Vaccine Technology Center of the Federal University of Minas Gerais Belo Horizonte 31310-260 Minas Gerais Brazil; ^6^ Department of Biochemistry and Immunology Institute of Biological Sciences Universidade Federal de Minas Gerais Belo Horizonte 31270-901 Minas Gerais Brazil ufmg.br; ^7^ Department of Clinical Analysis Faculty of Pharmacy Universidade Federal de Minas Gerais Belo Horizonte 31270-901 Minas Gerais Brazil ufmg.br

**Keywords:** adjuvants, effectiveness, Emulsivant 70, Montanide ISA 70, safety

## Abstract

Adjuvants are usually a fundamental part of a vaccine formulation due to their ability to increase immunogenicity, but their safety and efficacy must be established in preclinical studies. In this sense, we evaluated Emulsivant 70, a new ready‐to‐use adjuvant based on water‐in‐oil (W/O) emulsion for veterinary vaccines. Its performance was compared with the commercial adjuvant Montanide ISA 70 in mice immunized with tetanus toxoid (TT) and inactivated influenza PR8. No substantial changes in renal or hepatic biochemical markers (urea, creatinine, alanine aminotransferase [ALT], and aspartate aminotransferase [AST]) were observed for any formulation. Emulsivant 70 induced lower reactogenicity at the injection site than Montanide ISA 70. Both adjuvants enhanced IgG and IgG1 responses, but Emulsivant 70 promoted a faster increase in total IgG for both antigens. IFN‐γ levels increased similarly across adjuvanted groups. Following the influenza A challenge, all PR8‐immunized were fully protected. Overall, this study provides the first evidence that Emulsivant 70 is a safe adjuvant that elicits less local reactivity and achieves immunogenicity comparable to Montanide ISA 70, while inducing a faster humoral response. These findings support Emulsivant 70 as a promising alternative for the development of veterinary vaccines.

## 1. Introduction

During the development of a vaccine, adjuvants can be added to the formulation to enhance and prolong the immune response, modulate response types, and reduce the immunization frequency and required antigen doses [1–3]. However, to be used as an adjuvant, they must ensure minimal adverse effects, high stability, and convenient administration in the species of interest [4]. Aluminum compounds are adjuvants widely recognized for their efficacy, safety, and tolerability [5], being commonly employed in human and veterinary vaccines [3, 5, 6]. On the other hand, emulsions and oil‐based adjuvants are frequently used in veterinary vaccines [4, 7], where their efficacy, relatively simple production, and cost‐ effectiveness make them particularly attractive for use in animal vaccines [6].

Emulsions are classified as oil‐in‐water (O/W) when oil droplets are dispersed in water; water‐in‐oil (W/O) when water droplets are dispersed in oil; or multiple emulsions, such as water‐in‐O/W (W/O/W) [5, 6]. Among emulsion‐based adjuvants, MF59 and AS03 are O/W emulsion systems included in the licensed vaccines for human use [8]. In veterinary medicine, several commercial emulsion‐based adjuvants are available. The Montanide ISA range includes adjuvants designed to form different types of emulsions, including W/O, O/W, and W/O/W systems, whereas Emulsigen and MetaStim are O/W emulsion adjuvants. These commercial adjuvants have been used in veterinary vaccine formulations containing different bacterial and viral antigens [6].

W/O emulsions are well known for eliciting strong and sustained adjuvant activity, although injection site reactions are relatively common with these formulations [1, 9]. Their potent immunostimulatory effect is primarily attributed to the depot mechanism, which enables the slow release of antigen at the injection site, crucial for maintaining antigen‐specific immune responses [10, 11].

Commercial W/O adjuvants such as Montanide ISA exemplify well‐established emulsion‐based systems widely used in veterinary vaccines. However, despite the availability of effective veterinary adjuvants, the development of new platforms remains relevant. Veterinary formulations must combine effective and long‐lasting immune responses while meeting requirements for safety, stability, ease of administration, manufacturing feasibility, and economic viability [4, 6]. Balancing these different requirements remains an important challenge in veterinary adjuvant development [6]. In this context, the development of new emulsion formulations capable of maintaining robust adjuvant activity while improving their safety profile remains relevant in veterinary vaccine innovation [12].

To advance the development of a new adjuvant system, this study evaluated Emulsivant 70 (CRODA, Brazil) [13] in an in vivo preclinical model, directly comparing its performance with Montanide ISA 70 (SEPPIC, France). Both adjuvants were formulated as W/O emulsions to enable a comprehensive assessment of safety, immunogenicity, and protective efficacy. The formulations were evaluated using antigens of distinct composition and complexity: a purified bacterial‐derived protein antigen (tetanus toxoid, TT) and a whole inactivated viral antigen (PR8 influenza virus; PR8‐IIV and PR8).

As indicators of immunogenicity, the antigen‐specific serum IgG isotype profile and IFN‐γ production by stimulated splenocytes from vaccinated animals were evaluated. In the PR8 model, mice were challenged, and protective efficacy was assessed by measuring viral load in lung tissue following infection. Local tissue integrity at the injection site, together with renal and hepatic biochemical markers, served as indicators of tolerance and toxicity. Overall, the findings demonstrate that Emulsivant 70 is a safe veterinary adjuvant, showing efficacy comparable to Montanide ISA 70 while inducing a faster humoral immune response and reduced local reactivity.

## 2. Materials and Methods

### 2.1. TT and Inactivated Influenza PR8 Antigen

The purified TT antigen was obtained from the Bio‐Manguinhos bacterial vaccine production facility (Fiocruz, Rio de Janeiro, Brazil) and consists of two disulfide‐linked chains of 100 and 50 kDa, with a monomer purity greater than 80%. PR8 viral particles were generated from sub‐confluent co‐cultures of HEK 293T and MDCK cells maintained in Dulbecco’s modified Eagle’s medium (DMEM) supplemented with 10% fetal bovine serum (FBS). Cells were cotransfected with plasmids encoding the eight genomic segments of the PR8 influenza virus and cultured in DMEM containing 1 µg/mL TPCK‐treated trypsin. PR8 viral seeds generated from cotransfected cell cultures were propagated by inoculating 10‐to‐11‐day‐old specific pathogen‐free (SPF) embryonated chicken eggs via the allantoic cavity. The eggs were incubated at 37°C for 48–72 h and subsequently chilled overnight at 4°C before harvesting the allantoic fluid. The collected allantoic fluid was clarified by low‐speed centrifugation and aliquoted. Viral stocks were characterized and quantified by the plaque assay in MDCK cells using an agarose overlay. Viral genotypes were confirmed by PCR and sequencing. Characterization of TT and PR8 by SDS‐PAGE and ELISA is shown in Figure S1.

The MDCK cell line (American Type Culture Collection [ATCC] CCL‐34, RRID: CVCL_0422), derived from female *Canis lupus familiaris* kidney epithelial cells, and the HEK‐293T cell line (ATCC CRL‐3216, RRID: CVCL_0063), derived from female *Homo sapiens* embryonic kidney cells expressing the SV40 large T antigen, were obtained from the ATCC. Experiments were performed using early‐passage cells after thawing. Cell line authentication was performed by the supplier prior to distribution using standard quality control procedures, including species verification and short tandem repeat (STR) profiling (for HEK‐293 T). According to ATCC documentation, the cell lines, neither MDCK nor HEK‐293 T, are listed among commonly misidentified or cross‐contaminated cell lines. No additional in‐house authentication was therefore performed. Both cell lines were routinely tested for *Mycoplasma* contamination by PCR using MGSO and GPO3 primers targeting a conserved region of the *Mycoplasma* 16S rRNA gene (270–300 bp). DNA was extracted from culture supernatants using the NucleoSpin kit (Macherey‐Nagel) according to the manufacturer’s instructions. All cultures used in the experiments tested negative for *Mycoplasma* contamination.

### 2.2. Animals and Experimental Groups

The experiments were carried out in accordance with the UFMG Ethics Committee on Animal Experimentation (CEUA/UFMG No. LW16/20), the ARRIVE guidelines, and EU Directive 2010/63/EU. Female SPF BALB/c mice (6–8 weeks old) were used. Mice were housed in a ventilated, pathogen‐free room (25 ± 1°C, with a 12‐h light/dark cycle) and were given food and water ad libitum. For both studies (TT and PR8), animals were randomly assigned into five treatment groups: (1) phosphate‐buffered saline (PBS) loaded with antigen; (2) antigen plus aluminum hydroxide (Alhydrogel 2%, Croda, Denmark, Frederikssund, Denmark) and CpG (B344, positive control); (3) W/O emulsion adjuvanted with Emulsivant 70 without antigen; (4) W/O emulsion adjuvanted with Emulsivant 70 loaded with the antigen; (5) W/O emulsion was adjuvanted with Montanide ISA 70 and loaded with antigen.

In the TT experiment, five groups (*n* = 8) received primary immunization on day 0, a booster on day 21, and were euthanized on day 51. On day 51, immediately before euthanasia, mice were anesthetized by subcutaneous administration of a ketamine/xylazine mixture (90.0 mg/kg ketamine and 8.0 mg/kg xylazine) at a site distinct from the intramuscular vaccination site. After confirmation of deep anesthesia (third anesthetic plane), euthanasia was performed by cervical dislocation in accordance with accepted ethical guidelines for the humane use of laboratory animals. Blood samples were collected on days 0, 21, and 51 to evaluate immune responses and renal/hepatic biomarkers, while tissue integrity at the injection site was assessed at the end of the study (Figure 1).

**Figure 1:**
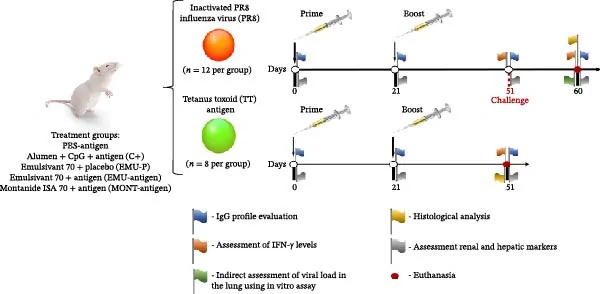
Timeline of the two immunization protocols (tetanus toxoid [TT] and inactivated influenza virus [PR8]).

In the PR8 experiment, five groups (*n* = 12) received a primary dose on day 0 and a booster on day 21. On day 51, four mice per group were euthanized for splenocyte culture, and the remaining eight mice per group were challenged with the influenza virus on day 51, followed by euthanasia on day 60. Blood samples were collected on days 0, 21, 51, and 60 to evaluate immune responses and renal/hepatic biomarkers. Tissue integrity at the injection site and lung viral load were assessed at the end of the study (Figure 1). For the influenza challenge, mice were anesthetized on day 51 with a subcutaneous injection of ketamine/xylazine (90 mg/kg ketamine and 8 mg/kg xylazine in PBS) at a site distinct from the intramuscular vaccination, followed by intranasal inoculation of 35 µL of PR8 viral suspension in PBS (pH = 7.4, 10 × LD_50_). On day 60, the mice were again anesthetized by subcutaneous injection before euthanasia.

All animals were vaccinated i.m. (100 µL) in the caudal dorsal region overlying the lumbosacral/sacral area. Blood samples were collected from the jugular vein, yielding ~150 µL per mouse, and transferred to sterile 500‐µL tubes for serum preparation and subsequent analyses. Animals were monitored after each administration for clinical and behavioral changes, and the body weight was recorded throughout the experiment. Relative body weight was calculated based on measurements obtained on day 1.

### 2.3. Vaccine Formulations

Formulations (10 µg TT or PR8/100 µL PBS) were prepared under controlled conditions (laminar flow hood) and assessed for sterility. For each immunization, a new laboratory batch was prepared. Briefly, PBS solutions (pH 7.4) were prepared, sterilized (127°C, 15 min), and filtered (Whatman GD/X syringe filters, polyethersulfone membrane, pore size 0.2 μm, diam. 25 mm, sterile) and cooled (5–6°C, ice bath) before antigen addition under constant homogenization (Ultra Turrax T‐25, Ika Labortechnik, Staufen, Germany). Homogenization (13,500 rpm) was maintained until the solution reached 36°C. A study was conducted during standardization to certify antigen stability, ensuring dose consistency for the preclinical study.

To produce Emulsivant 70 W/O emulsions with antigens (EMU‐TT and EMU‐PR8) and blank (EMU‐P), the aqueous (AP, PBS buffer) and oil (OP, Emulsivant 70) phases were prepared separately. AP (PBS solution, 30%) was prepared as described above, while OP (70% of Emulsivant 70 W/O emulsion) was composed of Emulsivant 70 filtered at 0.22 µm (Whatman GD/X syringe filters, polyethersulfone membrane, diam. 25 mm, sterile). After cooling both phases (5–6°C, ice bath), AP (with or without antigen) was added dropwise to the OP under stirring (Ultra Turrax T‐25, 13,500 rpm). Stirring was maintained until emulsions reached 36°C. Emulsions produced with Montanide ISA 70 W/O (MONT‐TT and MONT‐PR8, 30% AP plus 70% Montanide ISA 70) were prepared following the same protocol. The viscosity analysis and micro‐ and macroscopic appearance of produced emulsions were monitored over 30 days: initial (D1), 15 days (D15), and 30 days (D30). Details of these characterizations are provided in the Supporting Information.

The positive control (C+) was obtained by simultaneously homogenization (500 rpm, 1 h, 25°C) of the antigen (PBS‐TT or PBS‐PR8, 10 µg/dose) with 30% v/v Alhydrogel 2% (Croda, Denmark, Frederikssund, Denmark), followed by the addition of CpG B344 (18 µg/dose) (500 rpm, 1 h, 25°C) for a total volume of 100 µL/dose.

### 2.4. ELISA for Immunoglobulins

Indirect ELISA was used to quantify antigen‐specific IgG isotype (IgG1and IgG2a), and the IgG2 a/IgG1 ratio was subsequently calculated. Goat anti‐mouse HRP‐conjugated antibodies (total IgG 1030‐05, IgG1 1070‐05, and IgG2a 1081‐05, Southern Biotech, Birmingham, Alabama, USA) were used. Corning polystyrene high‐binding 96‐well microplates (Stripwell, Kennebunk, Maine, USA) were coated overnight (2–8°C) with 100 μL/well of TT or PR8 antigen (500 ng/well) in carbonate buffer (pH = 9.0). Plates were then washed once with PBS containing 0.05% Tween 20 and blocked with 250 μL/well of 2.5% skimmed milk in the washing solution for 2 h at 25°C.

ELISA conditions were standardized prior to analyses, and a 1:100 serum dilution was selected for providing a consistent and discriminative response profile. Serum samples (1:100 in PBS, pH = 7.4, supplemented with 2.5% skimmed milk) were added to TT/PR8‐coated plates and incubated for 1 h at 37°C, followed by four washes with wash solution (100 mM sodium phosphate dibasic, 18 mM sodium phosphate monobasic, 1500 mM sodium chloride, 28 mM potassium chloride, 1% w/v Tween 80, 0.1% v/v ProClin 300, and water for injections to make 1500 mL of solution).

HRP‐conjugated secondary antibodies (total IgG and IgG1 at 1:40,000; IgG2 at 1:10,000; diluted in blocking solution) were added (100 μL/well), and plates were incubated for 1 h at 37°C, followed by four washes. Then, 100 μL/well of 3,3^′^,5,5^′^‐tetramethylbenzidine substrate (TMB, Ultra‐Sensitive, Life Technologies) was added, and the plates were incubated for 15 min (25°C). The reaction was stopped with 0.5M H_2_SO_4_ (100 μL/well), and absorbance was measured at 450 nm using a Multiskan GO microplate reader (Thermo Scientific, Waltham, Massachusetts, USA). OD_450_ values were used as a comparative measure of relative antigen‐specific antibody responses.

### 2.5. Evaluation of IFN‐γ Cytokine in Splenocytes Culture

To assess IFN‐γ production, splenocyte cells collected on day 51 from each experimental group (TT: eight mice/group; PR8 prechallenge: four mice/group; PR8 postchallenge: eight mice/group) were cultured as described in the Supporting Information and stimulated with concanavalin A (ConA), sterile saline (WS ‐ without stimulus), PR8, and TT, which were previously diluted to 10 µM in RPMI + 10% FBS. Cultures were incubated (5% CO_2_, 37°C, 48 h), centrifuged, and supernatants stored at −80°C until the cytokine analysis via cytometric bead array (CBA). CBA followed the BD CBA Mouse Th1/Th2/Th17 Cytokine Kit protocol (Becton Dickinson, Franklin Lakes, New Jersey, USA), using 25 µL of supernatant with all available cytokine beads in it, as detailed in the Supporting Information. Readings were performed on a BD FACS Verse Flow Cytometer (Becton Dickinson, Franklin Lakes, New Jersey, USA), and data were analyzed using BD FCAP software (Becton Dickinson, Franklin Lakes, New Jersey, USA).

### 2.6. Assessment of Renal and Hepatic Markers

The safety of the formulations was evaluated based on biochemical analyses (Bioclin Quibasa, Belo Horizonte, Minas Gerais, Brazil). The main renal (creatinine and urea) and hepatic (aspartate aminotransferase [AST] and alanine aminotransferase [ALT]) markers were evaluated in the serum of the mice.

### 2.7. Assessment of Viral Load in Lung Tissue

For viral load determination, MDCK cells were cultured in DMEM (Gibco, Renfrew, UK) supplemented with 10% FBS (Gibco, Renfrew, UK) and 1% penicillin–streptomycin (Sigma–Aldrich, St. Louis, USA) in 6‐well plates (1.3 × 10^6^/well). The right lung lobe from infected mice was macerated (200 mg tissue/mL PBS) for 5 min at 50 Hz using a TissueLyser LT (Qiagen, Germantown, MD, USA) and centrifuged (10 min, 10,000 × *g*, 4°C). Supernatants were serially diluted in low‐glucose DMEM (up to 10^−6^). Subsequently, the supernatant of MDCK cells was removed, and 400 μL of diluted lung homogenate was added per well, followed by incubation for 1 h at 37°C. A/PR/8/34 virus (10^−6^ dilution) served as the positive control, and uninfected MDCK cells as the negative control (mock). After incubation, the inoculum was removed and replaced with prewarmed DMEM containing 1% agarose, 0.3% BSA, and 2 µg/mL trypsin (TPCK, Sigma–Aldrich, Saint Louis, USA). Plates were incubated for 72 h at 37°C with 5% CO_2_ to allow plaque formation. Cells were then fixed with 4% formaldehyde and 1% crystal violet (15 min), and viral load was quantified by counting plaque‐forming units (PFU) using Equation (1):

(1) PFU well0.4 mL×dilution factor×1.5 mL DMEM0.15 g of tissue=PFUg of tissue.

### 2.8. Histological Analysis at the Application Site

After euthanasia of the mice, tissue samples from the site of application of the formulations were collected and fixed in 10% buffered formalin (48 h) for histological analysis. The tissues were processed in the Lupetec processor (Model PT05, Lupe LTDA, São Carlos, São Paulo, Brazil), embedded in paraffin (60°C), sectioned (4 μm), stained with hematoxylin and eosin (H&E), and mounted on slides. The images were digitized using a Leica DM500 microscope (Leica Application Suite software, version 2.4.1, Leica, Wetzlar, Germany). A pathologist performed the histological analyses in the “single‐blind” model, and the inflammatory reaction was assessed semiquantitatively by the presence of leukocytes in the dermis and hypodermis. The inflammatory infiltrate scoring system was adapted from Alves et al. [14], as follows: 0 = absent, without mononuclear cell exudate of (histologically normal dermis); 1 = slight, diffuse mononuclear exudate in the upper dermis (1–9 cells per field/20 fields); 2 = moderate, diffuse or focal mononuclear exudate around vessels, glands, in the deep dermis or hypodermis (10–30 cells per field/20 fields); 3 = intense, diffuse or severe focal mononuclear exudate around vessels, glands in the deep dermis or hypodermis (>30 cells per field/20 fields); 4 = very intense, severe, and generalized inflammatory infiltrate throughout the dermis.

### 2.9. Statistical Analysis

Data were expressed as the mean ± standard error of the mean (SEM). Normality and homoscedasticity were analyzed by the Kolmogorov–Smirnov and Brown–Forsythe tests, respectively. Parametric data were analyzed by one‐way ANOVA, followed by Tukey’s post hoc test, whereas non‐parametric data were evaluated using the Kruskal–Wallis test with Dunn’s post hoc test. Statistical analyzes were performed using GraphPad Prism Version 9.02 (GraphPad Software Inc., San Diego, California, USA). Changes in relative body weight and renal and hepatic biochemical markers were evaluated descriptively and were not subjected to inferential statistical analysis.

## 3. Results

### 3.1. Formulations and Physical–Chemical Characterization

Figure 2 shows EMU‐P, EMU‐PR8, and MONT‐PR8 emulsions. After production, formulations were milky, opaque, with low viscosity and no phase separation (Figure 2A,F,K). At D15 (Figure 2B,G,L) and D30 (Figure 2C,H,M), oil release and sedimentation were observed in all formulations when evaluated under light but without emulsion breakage or water release (Table S1 and Figure S4). When evaluated under the microscope, Emulsivant 70 emulsions presented larger globules (Figure 2I,N) than Montanide ISA 70 emulsions (Figure 2D), maintaining this profile at 4°C for 30 days. All emulsions showed a Newtonian fluid profile (Figure 2E,J,O) with little variation in viscosity. Over 30 days, the viscosity of EMU‐P varied from 34.7 to 37.7 Cp, that of EMU‐PR8 from 35.6 to 37.3 Cp, and that of MONT‐PR8 from 39.1 to 43.5 Cp, being slightly higher. A summary of these results is presented in Table S1 and Figure S4.

**Figure 2:**
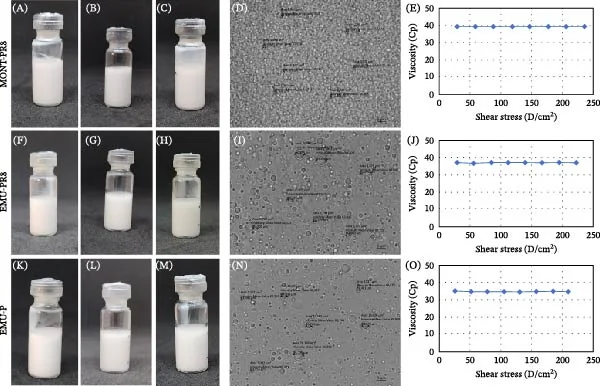
Characteristics of EMU‐P, EMU‐PR8, and MONT‐PR8 emulsions. (A) MONT‐PR8 on day 1 (D1). (B) MONT‐PR8 on day 15 (D15). (C) MONT‐PR8 on day 30 (D30). (D) MONT‐PR8 globules on D30. Bar = 5 µm. (E) Rheological profile of MONT‐PR8 on D30. (F) EMU‐PR8 on D1. (G) EMU‐PR8 on D15. (H) EMU‐PR8 on D30. (I) EMU‐PR8 globules on D30. Bar = 5 µm. (J) Rheological profile of EMU‐PR8 on D30. (K) EMU‐P on D1. (L) EMU‐P on D15. (M) EMU‐P on D30. (N) EMU‐P globules on D30. Bar = 5 µm. (O) Rheological profile of EMU‐P on D30.

### 3.2. Immune Response Against TT

Figure 3 shows antibody responses before and after immunization and cytokines assessment post‐vaccination. After the prime vaccination (Figure 3A), EMU‐TT induced higher IgG responses than PBS‐TT (*p* < 0.0001) and MONT‐TT (*p* < 0.0001), with levels comparable to C+(TT) (*p* = 0.1844). After boosting, EMU‐TT, MONT‐TT, and C+(TT) were statistically similar (*p* > 0.8300), all inducing higher IgG levels than PBS‐TT (*p* < 0.0451).

**Figure 3:**
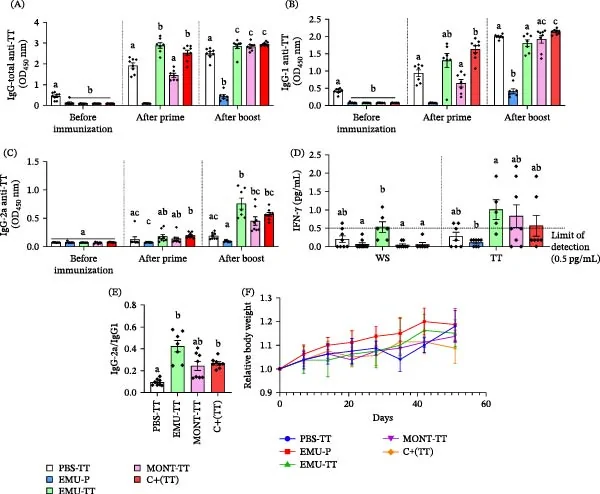
Immune responses and body weight of mice, before and after immunization with tetanus toxoid antigen (TT) formulations. (A) Total IgG responses. (B) IgG1 responses. (C) IgG2a responses. (D) IFN‐γ production by splenocytes after TT stimulation or without stimulation (WS). (E) IgG2 a/IgG1 ratio measured after the booster dose. (F) Relative body weight of mice throughout the study. Data are from one independent in vivo experiment comprising five experimental groups (*n* = 8 mice/group; *N* = 40 total) and are presented as mean ± SEM. Statistical analyses were performed as described in Section 2.9, using one‐way ANOVA followed by Tukey’s post hoc test or Kruskal–Wallis followed by Dunn’s post hoc test, as appropriate. Different lowercase letters indicate statistically significant differences among treatment groups within the same time point (*p* < 0.05); groups sharing at least one letter are not significantly different. For IFN‐γ production, comparisons were performed among treatment groups separately within the nonstimulated and TT‐stimulated conditions. Exact *p*‐values for the relevant comparisons are reported in Section 3.

Postprime IgG1 responses (Figure 3B) were similar among animals immunized with EMU‐TT, MON‐TT, and PBS‐TT (*p* > 0.7914). Notably, only EMU‐TT elicited IgG1 levels statistically equivalent to the positive control C+(TT) (*p* > 0.9999), in which TT was formulated with Alhydrogel and CpG, two adjuvants known to enhance vaccine immunogenicity [15, 16]. After the boost, IgG1 responses increased in all groups, with EMU‐TT and MONT‐TT being statistically similar to PBS‐TT (*p* > 0.3734), and MONT‐TT also did not differ from C+(TT) (*p* = 0.2439). IgG2a responses increased only after boosting (Figure 3C), being higher in EMU‐TT, which differed from PBS‐TT (*p* = 0.0079), but not from MONT‐TT or C+(TT) (*p*  > 0.9999). All adjuvanted formulations promoted an IgG response biased towards IgG1 rather than IgG2a (Figure 3E). When IFN‐γ levels were compared with and without TT restimulation (Figure 3D), all groups showed undetectable cytokine levels in the absence of the antigen. TT restimulation induced basal IFN‐γ levels, with no significant differences among EMU‐TT, MONT‐TT, C+(TT), and PBS‐TT. ConA stimulation increased IFN‐γ in MONT‐TT, EMU‐TT, and C+(TT), confirming the assay responsiveness (Figure S2). Together, these results indicate that all adjuvanted groups generated humoral responses, whereas antigen‐specific IFN‐γ production remained low and did not differ significantly among treatments. Throughout the 51 days of the evaluation, the vaccinated animals remained healthy, eating and moving normally, with no deaths or clinical changes. Figure 3F illustrates the relative body weights of mice throughout the study, showing that immunizations did not affect the animals’ weight gain over time.

### 3.3. Immune Response Against Influenza

Figure 4 illustrates IFN‐γ levels in PR8‐vaccinated animals pre‐ and postchallenge. Before challenge, IFN‐γ levels were low or below the detection limit of the kit (0.5 pg/mL), even with PR8 stimulation (Figure 4A). After challenge with influenza A, IFN‐γ levels increased in all groups vaccinated with formulations containing PR8. Although some statistically significant differences were detected among treatment groups, these were limited to specific comparisons and did not reveal a consistent treatment‐related pattern in PR8‐specific responses (Figure 4B). ConA stimulation induced higher IFN‐γ production in MONT‐PR8, EMU‐PR8, and C+(PR8), consistent with its use as a positive stimulation control (Figure S3).

**Figure 4:**
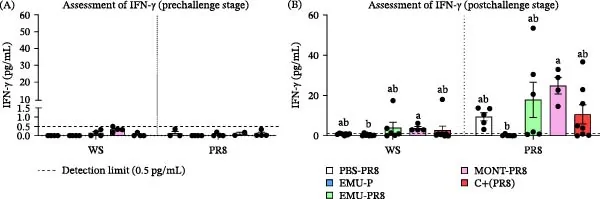
IFN‐γ production by splenocytes from vaccinated mice before and after challenge with PR8 influenza virus, following specific stimulation with PR8 or without stimulation (WS). (A) Prechallenge IFN‐γ levels (*n* = 4 mice/group). (B) Postchallenge IFN‐γ levels (*n* = 8 mice/group). Data are from one independent in vivo experiment comprising five experimental groups (*n* = 12 mice/group; *N* = 60 total) and are presented as mean ± SEM. Statistical analyses were performed as described in Section 2.9, using one‐way ANOVA followed by Tukey’s post hoc test or Kruskal–Wallis followed by Dunn’s post hoc test, as appropriate. Comparisons among treatment groups were performed separately within the nonstimulated and PR8‐stimulated conditions. Different lowercase letters indicate statistically significant differences among treatment groups within the same condition (*p* < 0.05); groups sharing at least one letter are not significantly different. Exact *p*‐values for the relevant comparisons are reported in Section 3.

Figure 5 illustrates total IgG, IgG1, and IgG2a responses in animals vaccinated with EMU‐P, EMU‐PR8, MONT‐PR8, PBS‐PR8, or C+(PR8). After the prime, all groups except EMU‐P showed increased total IgG and IgG1 (Figure 5A,B). The EMU‐PR8 group showed higher total IgG (*p* < 0.0001) than MONT‐PR8 but did not differ significantly from PBS‐PR8 or C+(PR8) (*p* > 0.0582). At the prechallenge phase, total IgG levels were higher in EMU‐PR8 and MONT‐PR8 compared with PBS‐PR8 (*p* < 0.0026). In the postchallenge phase, adjuvanted groups (C+(PR8); EMU‐PR8 and MONT‐PR8) maintained higher total IgG, with only EMU‐PR8 differing statistically from PBS‐PR8 (*p* = 0.0005). Overall, no adjuvant‐related increase in total IgG levels was detected after the prime when compared with PBS‐PR8. However, EMU‐PR8 displayed an earlier increase in total IgG (*p* < 0.0001) compared with MONT‐PR8. After boosting, both EMU‐PR8 and MONT‐PR8 showed higher total IgG levels than PBS‐PR8 (*p* < 0.0026).

**Figure 5:**
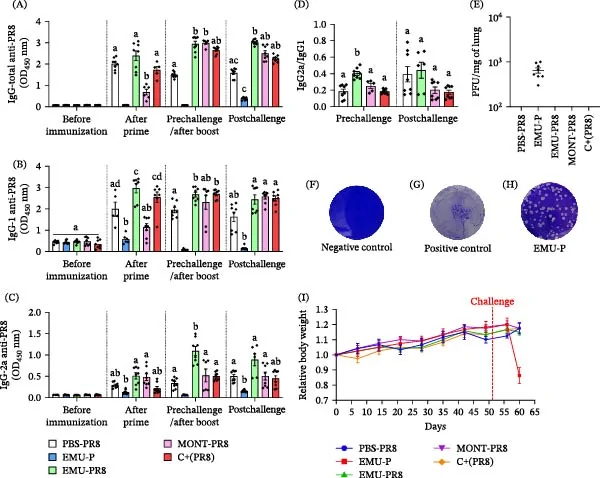
Immune responses and mice weight, before and after immunization with PR8 formulations; and pre‐ and postchallenge with influenza A. (A) IgG‐total. (B) IgG1. (C) IgG2a. (D) IgG2 a/IgG1 in pre‐ and postchallenge. (E) Viral loads in lung tissues postchallenge with influenza virus. (F) Plate’s well with uninfected MDCK cells (mock, negative control). (G) Plate’s well with MDCK cells infected with influenza virus culture (positive control). (H) Plate’s well with MDCK cells inoculated with supernatant from macerated lungs of mice vaccinated with placebo formulation (EMU‐P) and challenged with influenza virus. (I) Relative body weight of mice throughout the study. Data are from one independent in vivo experiment comprising five experimental groups (*n* = 12 mice/group; *N* = 60 total) and are presented as mean ± SEM. Statistical analyses were performed as described in Section 2.9, using one‐way ANOVA followed by Tukey’s post hoc test or Kruskal–Wallis followed by Dunn’s post hoc test, as appropriate. Different lowercase letters indicate statistically significant differences among treatment groups within the same evaluated period (*p* < 0.05); groups sharing at least one letter are not significantly different. Exact *p*‐values for the relevant comparisons are reported in Section 3. Relative body‐weight changes were evaluated descriptively.

Regarding IgG1 (Figure 5B), a similar pattern was observed. After the prime, EMU‐PR8 and C+(PR8) showed the highest IgG1 levels, with EMU‐PR8 differing from MONT‐PR8 (*p* < 0.0001) and PBS‐PR8 (*p* = 0.0349). The groups that were vaccinated with the adjuvanted formulations maintained high IgG1 levels until the end of the experiment. For IgG2a levels (Figure 5C), in the prechallenge phase, EMU‐PR8 induced a more pronounced increase compared to the other groups (*p* < 0.0456), but no statistical difference was observed in the postchallenge phase. The IgG2 a/IgG1 ratio in the pre‐ and postchallenge stage (Figure 5D) showed similar results to TT immunization, with all adjuvanted formulations promoting an IgG response biased towards IgG1 over IgG2a.

Figure 5E–H shows the viral load in the lungs after challenge with the influenza virus. Vaccination with formulations containing the antigen significantly reduced the viral load, with infected cells being observed only in animals vaccinated with EMU‐P (Figure 5E,H). Regarding the clinical follow‐up of the animals, no deaths were observed during the study, and the mice remained healthy, eating, and normal activity. Figure 5I shows the relative body weights, where only animals vaccinated with EMU‐P lost weight after the challenge with PR8. Without prior immunity to PR8, EMU‐P (without the antigen) failed to induce protection, leading to increased viral titer and weight loss.

### 3.4. Renal and Hepatic Markers

Figure 6A–D illustrates the results of the evaluation of biochemical markers in serum samples from mice in the TT study. As can be seen, urea levels were slightly elevated throughout the study in all groups, which demonstrates that this effect is not related to immunization or the adjuvant (Figure 6A). This alteration may be diet‐related as creatinine levels (Figure 6B) remained low and within the species’ reference range [17], suggesting no kidney damage. Hepatic markers (ALT and AST) were slightly elevated in all groups, probably due to sample hemolysis. In the mice studied with PR8 (Figure 7A–D), urea and creatinine levels remained within the species’ reference range [17]. Hepatic markers were also slightly elevated in all groups, again possibly due to hemolysis.

**Figure 6:**
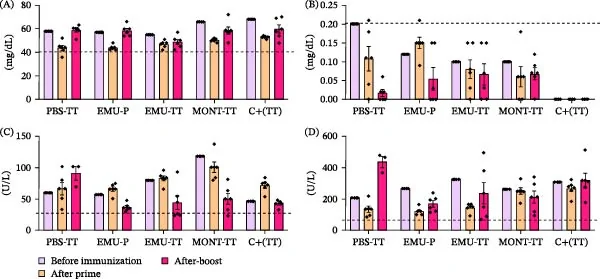
Evaluation of renal (A: urea and B: creatinine) and hepatic (C: ALT [alanine aminotransferase] and D: AST [aspartate aminotransferase]) biochemical markers in the serum of mice in the study with tetanus toxoid (TT). Data are from one independent in vivo experiment (*n* = 8 mice/group; *N* = 40 total) and are presented as mean ± SEM. Biochemical markers were evaluated descriptively.

**Figure 7:**
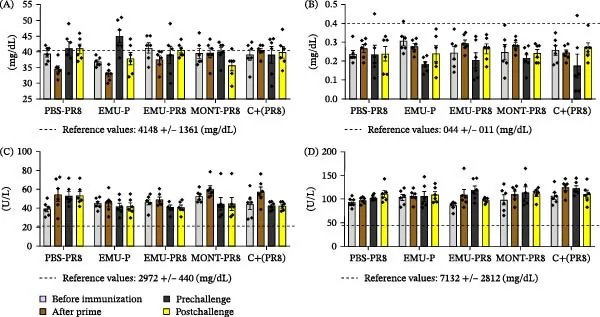
Evaluation of renal (A: urea and B: creatinine) and hepatic (C: ALT [alanine aminotransferase] and D: AST [aspartate aminotransferase]) biochemical markers in the serum of mice in the study with PR8. Data are from one independent in vivo experiment (*n* = 12 mice/group; *N* = 60 total) and are presented as mean ± SEM. Biochemical markers were evaluated descriptively.

### 3.5. Histological Analysis at the Application Site

Figure 8 shows the histological and inflammation responses at the vaccine application site. In TT‐vaccinated animals, skin histology revealed no lesions in the epidermis or dermis, with occasional alterations restricted to the dermis and hypodermis. However, alterations in deeper layers (adventitia, below the muscle) were observed in most groups. Figure 8A shows the inflammatory infiltrate scores in the dermis and adventitia of TT‐vaccinated animals. All adjuvanted formulations, including the placebo (EMU‐P), induced inflammatory infiltrates, with no significant differences among groups (*p* > 0.4649). The presence of these infiltrates is consistent with local inflammatory reactions previously reported for mineral oil‐based W/O emulsion adjuvants [4]. Figure 8B presents the inflammatory infiltrate scores in the dermis and adventitia of PR8‐vaccinated animals. The MONT‐PR8 group differed significantly from all other groups (*p* ≤ 0.0182), except from C+(PR8) (*p* = 0.1332). The EMU‐PR8 group exhibited higher scores than PBS‐PR8 (*p* = 0.0310) but did not differ statistically from EMU‐P (*p* = 0.9832) or C+(PR8) (*p* = 0.9435). Importantly, the histopathological assessment revealed distinct local tissue responses among the PR8 formulations. While inflammatory infiltrates were observed in EMU‐PR8, no necrosis‐like lesions were detected at the injection sites, in contrast to the MONT‐PR8 and C+(PR8) groups, where such lesions were present. This distinction is particularly relevant for the comparative evaluation of Emulsivant 70, as it demonstrates a more favorable local tolerability profile than the established commercial Montanide ISA 70 while preserving immunogenicity and protective efficacy. The absence of necrosis‐like lesions in the Emulsivant 70 group, despite the expected local inflammatory response associated with adjuvant activity, represents a meaningful safety advantage, supporting its continued development as a veterinary vaccine adjuvant.

**Figure 8:**
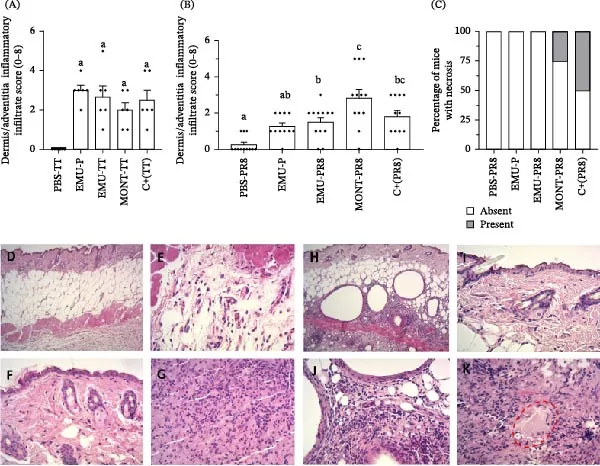
Inflammatory infiltrate score in the dermis/adventitia, percentage of necrosis, and representative histological aspects of the skin at the vaccine application site. (A) Inflammatory infiltrate score in the dermis/adventitia of mice from the TT study. Histological analysis was performed in all animals from the experiment (one independent in vivo experiment; *n* = 8 mice/group; *N* = 40 total). (B) Inflammatory infiltrate score in the dermis/adventitia of mice from the PR8 study. Histological analysis was performed in all animals from the experiment (one independent in vivo experiment; *n* = 12/group; *N* = 60 animals). (C) Percentage of necrosis in mice from the PR8 study, based on the histopathological assessment of all animals. (D–G) Representative histological images of the skin at the site of application of the formulation with C+(PR8) at the prechallenge stage. Magnification = 100x (D, E) and 400x (F, G). (H–K) Representative histological images of the skin at the site of application of the formulation with MONT‐PR8 at the postchallenge stage. Magnification = 100x (H) and 400x (I–K). The histopathological scores were determined from the analysis of all animals in each experimental group, whereas representative images are shown for illustrative purposes. Data are derived from the two independent in vivo experiments conducted with TT and PR8 and are presented as mean ± SEM. Statistical analyses were performed as described in Section 2.9, using one‐way ANOVA followed by Tukey’s post hoc test or Kruskal–Wallis followed by Dunn’s post hoc test, as appropriate. Different lowercase letters indicate statistically significant differences among experimental groups within the same evaluated condition (*p* < 0.05); groups sharing at least one letter are not significantly different. Exact *p*‐values for the relevant comparisons are reported in Section 3.

Macroscopic analysis of the injection site showed that the emulsions were not completely absorbed, which was expected given the presence of mineral oil in the composition of the adjuvants Emulsivant 70 and Montanide ISA 70. No skin changes were observed in mice vaccinated with EMU. However, in mice vaccinated with C+(PR8) and MONT‐PR8, small foci of tissue necrosis were detected at the injection site (Figure 8C). In the C+(PR8) group, a larger, slightly eosinophilic mass was observed in the adventitial layer, suggestive of necrosis. This area was surrounded by significant mixed infiltration, predominantly neutrophils (Figure 8D–F). In Figure 8G, a neutrophilic infiltrate is observed in the adventitial region, with inflammation largely composed of neutrophilic cells. In the MONT‐PR8 group, postchallenge histology revealed substantial leukocyte infiltration in both the dermis and adventitia, as well as capsular structures lined by epithelium in the adventitia (Figure 8H–I). Furthermore, neutrophils and cells with pyknotic nuclei, which are indicative of cell death, were also observed (Figure 8J). In Figure 8K, an amorphous eosinophilic mass can be seen, surrounded by tissue in a degenerative process and more externally surrounded by fibroblasts and leukocytes.

## 4. Discussion

Although established veterinary adjuvants are available, the development of new systems remains a market demand driven by the need for safer, more effective, stable, and economically viable products. In this context, we compared Emulsivant 70, a novel veterinary adjuvant, with the widely used Montanide ISA 70 through formulation, safety, and efficacy studies employing two structurally distinct antigens, TT and PR8. The results demonstrated an improved safety profile for Emulsivant 70, along with faster humoral responses and efficacy comparable to Montanide ISA 70, providing initial evidence that Emulsivant 70 is a safe and effective alternative adjuvant for veterinary vaccines. Having established these overall findings, the detailed results are discussed below, beginning with the physicochemical characteristics of the formulations.

The incorporation of Emulsivant 70 or Montanide ISA 70 into W/O emulsions resulted in stable emulsions, with only slight oil release and sedimentation observed, without emulsion breakage, an essential stability criterion [10]. Although the viscosity of the emulsion with Montanide ISA 70 was slightly higher, it remained below 250 Cp, being suitable for injection [18]. Regarding the size of the globules, in the emulsions with Emulsivant 70, the globules were larger, but this did not negatively affect any of the evaluated efficacy or safety parameters. The correlation between immune response and globule/particle size remains inconclusive, with studies reporting variable outcomes [6, 18–21].

In the TT study, antigen‐specific restimulation induced only basal IFN‐γ production, with no statistically significant differences among treatment groups. Similarly, in the PR8 study, no relevant PR8‐specific IFN‐γ production was detected after the immunization period, including in animals receiving Emulsivant 70 or Montanide ISA 70. Under the experimental conditions evaluated, neither the candidate adjuvant nor the established commercial W/O adjuvant elicited a measurable antigen‐specific IFN‐γ response before viral challenge. This is an important observation for the comparative assessment of the adjuvants as it indicates no differential effect of Emulsivant 70 or Montanide ISA 70 on this cellular immune parameter.

IFN‐γ is a hallmark of Th1‐type immunity and contributes to antiviral defense and protection against several infectious diseases [22, 23]. However, previous studies have reported variable effects of Montanide ISA 70 on IFN‐γ responses depending on the antigen and experimental model. Jang et al. [24] observed increased IFN‐γ gene transcription in chickens immunized with recombinant *Eimeria acervulina* profilin using Montanide ISA 70 VG, whereas Jawale et al. [25, 26] reported no significant differences in IFN‐γ mRNA expression or protein production between groups immunized with or without Montanide ISA 70 VG. These findings support the notion that IFN‐γ responses induced by W/O adjuvants are highly dependent on the antigenic context and experimental conditions.

Following the PR8 challenge, IFN‐γ production increased relative to prechallenge levels in the PR8‐immunized groups, consistent with immune activation triggered by viral exposure. Although some statistically significant pairwise differences were detected postchallenge, these were limited to specific comparisons and did not reveal a consistent pattern attributable to either Emulsivant 70 or Montanide ISA 70. Thus, the postchallenge rise in IFN‐γ should be interpreted primarily as a response to viral infection rather than as evidence of differential cellular priming induced by the adjuvants. Because no relevant PR8‐specific IFN‐γ response was detected after immunization with either adjuvant, the present design does not allow the postchallenge IFN‐γ response to be clearly attributed to vaccine‐induced cellular memory. Within this comparative framework, the absence of a differential IFN‐γ response between Emulsivant 70 and Montanide ISA 70 indicates comparable performance for this cellular immune parameter.

Regarding total IgG levels, at the end of both the TT study and PR8 studies, all groups vaccinated with adjuvanted formulations showed higher levels of total IgG. However, Emulsivant 70 induced a faster IgG production (after 1st dose) in both TT and PR8 studies, outperforming Montanide ISA 70 and showing similar results to the C+ (Alhydrogel plus CpG). In the PR8 study, EMU‐PR8 was the only group with total IgG levels statistically higher than PBS‐PR8 at the end of the experiment.

Substantial total IgG responses were also observed when antigens were administered in PBS alone, consistent with the known immunogenicity of whole inactivated viruses, such as PR8, and highly immunogenic protein antigens, such as TT [27, 28]. However, adjuvants can enhance the magnitude, speed, and durability of these responses [29, 30]. This was reflected in the statistically higher total IgG levels observed for EMU‐TT after prime and boost and for EMU‐PR8 after boost and postchallenge.

Accelerated induction of antibody responses is a valuable strategy in vaccine development, particularly when early protection is required for disease control [31]. It is important to highlight that Montanide ISA 70 has been widely validated as an effective IgG‐inducing adjuvant in diverse models, including vaccines against fowl typhus [26], *Leishmania major* [32], mastitis (*Staphylococcus aureus*) [33], avian coccidiosis [24], and avian salmonellosis [25]. Thus, the earlier IgG response induced by Emulsivant 70 compared with Montanide ISA 70, an established benchmark adjuvant, supports its potential for vaccine optimization.

Regarding IgG isotypes, Emulsivant 70 induced high IgG1 levels, similar to Montanide ISA 70 and Alhydrogel + CpG. All adjuvants increased IgG2a responses against TT compared to PBS‐TT. In the PR8 study, before challenge, Emulsivant 70 induced a more pronounced IgG2a response than the other groups (*p* < 0.0456), indicating a stronger IgG2a component at this time point. IgG2a antibodies have been associated with better viral clearance and protection [34, 35]. However, the IgG2 a/IgG1 ratio remained <1 across all immunized groups for both TT and PR8, indicating a predominantly Th2‐associated humoral response [36]. This is consistent with previous studies in BALB/c mice, where subunit and inactivated influenza vaccines typically induce predominantly Th2‐immune responses [35, 37, 38]. In the influenza challenge, protection was assessed based on the infectious viral load in lung tissue. All groups immunized with PR8 developed protective immunity as no viral load or deaths were recorded. The virus was detected only in the placebo group (EMU‐P), which also showed marked weight loss. As expected, these mice had no prior immunity to PR8, and treatment with EMU‐P (without the antigen) did not induce an immune response. Although adjuvants are known to potentiate the immune response, making vaccination more efficient than prior contact with the pathogen [39, 40], prior contact may confer some protection [41]. In the study by Martin et al. [34], naïve mice lost 25% of their body weight after challenge with 5× LD_50_ and died on days 5–6 when challenged with 20× LD_50_, whereas 50% of mice vaccinated with inactivated PR8 and ~100% of those vaccinated with adjuvanted formulations survived. Protective efficacy has been demonstrated in challenge models using vaccines formulated with Montanide ISA 70 or ISA70VG, including vaccines against *Trichinella* [42], fowl typhus [26], Newcastle Disease [43], and avian influenza [44].

Under the experimental conditions used in the present study, the challenge model did not discriminate a protective benefit of the adjuvanted formulations over PR8 administered without the adjuvant since all antigen‐immunized groups were protected. As this is the first preclinical evaluation of Emulsivant 70 as a vaccine adjuvant, further studies are planned to expand its characterization, including dose optimization, evaluation with additional antigen types, pulmonary histopathological analysis, and challenge models capable of better discriminating differences in disease severity and survival among formulations.

Regarding the safety of the vaccine formulations evaluated, histological analyses showed exacerbated inflammatory reactions and necrosis‐like changes in the adventitial layer only in animals vaccinated with MONT‐PR8 and C+(PR8). In line with these findings, Marcq et al. [45] reported that chicken immunized with Montanide ISA 70 VG exhibited injection site reactions ranging from mild inflammatory infiltrate to granulomatous inflammation and hyaline necrotic areas. In rabbits and mice, Montanide ISA 50 (85% mineral oil and 15% mannide oleate) also caused tissue changes [46], but less severe than those observed for Freund’s adjuvant (FA). These reports indicate that local inflammatory reactions are not unexpected following administration of mineral oil‐based W/O emulsion adjuvants [4]. Johnston et al. [47] observed that Montanide ISA 50 induced lesions comparable to FA in rabbits.

Taken together, the histopathological findings support a superior local tolerability profile for Emulsivant 70 compared with the reference adjuvants. Necrosis‐like lesions were observed at the injection sites of animals receiving Montanide ISA 70 and Alhydrogel + CpG, whereas no lesions of this type were detected in the Emulsivant 70 groups despite the presence of local inflammatory infiltrates. Although both Emulsivant 70 and Montanide ISA 70 are mineral oil‐based emulsions, variations in their surfactant systems and physicochemical properties may influence local tissue tolerance [48, 49]. Moreover, the severity of local lesions may also depend on the antigenic context [46], which may help explain the absence of necrosis‐like changes in the TT‐immunized groups. Importantly, the absence of necrosis‐like lesions with Emulsivant 70, combined with its earlier humoral response relative to Montanide ISA 70 and its preserved protective efficacy in the PR8 challenge model, supports a favorable balance between immunogenicity, protection, and local tolerability. These attributes strengthen the potential of Emulsivant 70 as a promising candidate adjuvant for further investigation.

Evaluation of renal and hepatic markers provides additional information into formulation safety. Urea levels were normal in the PR8 study and slightly elevated across all groups in the TT study. Because the studies were conducted independently and all groups showed either normal (PR8 study) or slight changes (TT study) in urea levels, these findings are unlikely to be related to immunogens or adjuvants. As noted by Barbosa et al. [17] and Sodré et al. [50], urea levels may be affected by diet and should be evaluated together with creatinine. Since creatinine levels remained unchanged, there was no evidence of renal injury. For hepatic markers (ALT and AST), slight changes were observed across all groups, regardless of antigen (TT or PR8). These changes are likely attributable to the blood collection procedure, which may have led to erythrocyte lysis. Thus, for both TT and PR8 vaccines, Emulsivant 70 demonstrated a safety profile comparable to Montanide ISA 70 and Alhydrogel plus CpG, with only mild and explainable changes and no evidence of renal or hepatic damage.

## 5. Conclusions

This study provides evidence supporting the safety and efficacy of Emulsivant 70, a new veterinary adjuvant. In addition to demonstrating immunogenicity comparable to Montanide ISA 70, Emulsivant 70 caused fewer injection site reactions and induced a faster humoral immune response. Together, these findings position Emulsivant 70 as a promising alternative for the development of new veterinary vaccines.

## Author Contributions

**Caroline Dohanik:** software, formal analysis, investigation, data curation, writing – original draft preparation, writing – review and editing, visualization. **Leandro Gonzaga de Oliveira**: formal analysis, investigation, data curation, writing – original draft preparation, writing – review and editing. **Roberta Ondei and Dennis Christensen**: methodology, writing – review and editing. **Diego dos Santos Ferreira and Lucas Antônio Miranda Ferreira**: methodology. **Ricardo Tostes Gazzinelli**: conceptualization, methodology, project administration, resources and funding acquisition. **Ana Paula Fernandes and Gisele Assis Castro Goulart**: conceptualization, methodology, validation, formal analysis, data curation, writing – review and editing, supervision, project administration, resources and funding acquisition.

## Funding

This work was partially supported by the Brazilian agencies Fundação de Amparo à Pesquisa do Estado de Minas Gerais (FAPEMIG), Coordenação de Aperfeiçoamento de Pessoal de Nível Superior (CAPES), and Conselho Nacional de Desenvolvimento Científico e Tecnológico (CNPq), as well as by Croda (Grant SIEX/UFMG 302678).

## Disclosure

All stages of the study, including its design, data collection, and data analysis, were conducted independently and impartially by the researchers. All authors have read, approved, and agreed to the published version of the manuscript. This work was presented in part at the 8th International Symposium on Immunobiologicals, Rio de Janeiro, Brazil, 7–10 May 2024 (“Preclinical efficacy and safety studies of Emulsivant 70: a promising adjuvant for emulsified veterinary vaccines”) [51].

## Conflicts of Interest

Roberta Ondei and Dennis Christensen are employees of Croda. The other authors declare no conflicts of interest.

## Supporting Information

Additional supporting information can be found online in the Supporting Information section.

## Supporting information


**Supporting Information** Supporting Information is available online at Journal of Immunology Research. Supporting Information includes supporting methods describing the preparation of splenocyte cultures used for the evaluation of IFN‐γ cytokine production. This section also describes the methods used for the characterization and stability evaluation of emulsions over 30 days. The supporting results include the following: Figure S1: (A) Characterization of TT and PR8 antigens by SDS‐PAGE. TT antigen titration curves using positive and negative sera (B) and PR8 with positive serum (C) and negative serum (D). Table S1: Characteristics of the emulsions (1st dose), loaded or not with the PR8 antigen, over 30 days. Figure S2: Production of IFN‐γ in splenic cells of mice (vaccinated with different formulations with and without the TT antigen) after ConA stimuli. Figure S3: Production of IFN‐γ in splenic cells of mice (vaccinated with different formulations with and without the PR8 antigen) after ConA stimuli, prechallenge and postchallenge. Figure S4: Macroscopic characteristics of the emulsions (1st dose) loaded or not with the PR8 antigen on day 15 (D15) and day 30 (D30) at 4°C.

## Data Availability

The data presented in this study are available from the corresponding author upon reasonable request.
