# Supplementary material for: Preclinical Safety and Immunogenicity Characterization of Emulsivant 70: A Promising Candidate Adjuvant for Emulsified Veterinary Vaccines
Source: J Immunol Res. 2026 Nov 6;2026:7245322. doi: 10.1155/jimr/7245322 (PMC13743927; doi:10.1155/jimr/7245322)
Supplement: Supplementary file 1 [file JIMR-2026-7245322-s001.docx]

**Supporting Information**

Preclinical efficacy and safety studies of Emulsivant™ 70: a promising adjuvant for emulsified veterinary vaccines

Caroline Dohanik ^a,†^, Leandro Gonzaga de Oliveira ^b,†^, Roberta Ondei ^c^, Dennis Christensen ^d^, Diêgo dos Santos Ferreira ^a,e^ , Lucas Antônio Miranda Ferreira ^a^, Ricardo Tostes Gazzinelli ^e,f^, Ana Paula Fernandes ^e,g^ and Gisele Assis Castro Goulart ^a,e,^*

^a^	Department of Pharmaceutical Products, Faculty of Pharmacy, Universidade Federal de Minas Gerais, Belo Horizonte 31270-901, Minas Gerais, Brazil;

^b^	Detechta, BH-TEC, Belo Horizonte 31310-260, Minas Gerais, Brazil;

^c^ Croda do Brasil, Rua Croda, 580, Campinas 13054-710, São Paulo, Brazil;

^d^ Croda Pharma, Vaccine Adjuvant Systems R&D, Diplomvej 381, DK-2800 Lyngby, Denmark;

^e^	Vaccine Technology Center of the Federal University of Minas Gerais, Belo Horizonte 31310-260, Minas Gerais, Brazil

^f^	Department of Biochemistry and Immunology, Institute of Biological Sciences, Universidade Federal de Minas Gerais, Belo Horizonte 31270-901, Minas Gerais, Brazil;

^g^ Department of Clinical Analysis, Faculty of Pharmacy, Universidade Federal de Minas Gerais, Belo Horizonte 31270-901, Minas Gerais, Brazil;

*Corresponding author at: Av Antônio Carlos, 6627, Faculty of Pharmacy, Department of Pharmaceutics Products, Campus Pampulha – Universidade Federal de Minas Gerais, 31270-901. Belo Horizonte (MG) – Brazil. Tel.: +55 31 3499-6939; Fax: + 55 31 3499-6830. Email addresses: gacg@ufmg.br

† These authors contributed equally to this work.

**Supplementary Materials and Methods**

***Splenocytes Culture***

Splenocyte cultures were obtained from the spleens of euthanized mice, maintained in incomplete RPMI medium (without FBS) (0-4°C). Tissues were macerated and homogenized using the Tissue-Lyzer equipment (Qiagen, Hilden, Germany) (5 cycles of 3 min. with 40 oscillations/minute), and then filtered, added with 3 mL of RPMI medium and centrifuged (1200 rpm for 10 min. at 4°C). After discarding the supernatant, the splenic cells were resuspended in BD erythrocyte lysis buffer (Becton Dickinson, Franklin Lakes, New Jersey, USA) (1.0mL), incubated in ice bath (5 min.) and neutralized with 2.0 mL of complete RPMI medium (RPMI supplemented with 10 % FBS). A new centrifugation was performed (1200 rpm, 5 min, 4°C) and, after discarding the supernatant, the resuspension with complete RPMI (3.0mL) and centrifugation process was repeated as previously specified. Finally, after discarding the supernatant, the cells were resuspended in complete RPMI (1.0 mL), and counting was performed using vital dye. The cell concentration was adjusted to 10^7^ cells/ml and was seeded per well.

***Cytometric Bead Array (CBA)***

The samples were acquired in a flow cytometer using an Instrument Software Template provided by BD (available at https://www.bdbiosciences.com/en-br/products/reagents/immunoassays/cba.) for bead gating strategy and analyzed with the BD FCAP Array software containing the appropriate bead library configurations for the CBA kit. The cytokine-specific bead populations were differentiated according to their fluorescence characteristics, and the fluorescence intensity associated with each analyte was converted into cytokine concentration using the corresponding standard curves generated by the software.

***Characterization and evaluation of emulsion stability***

Emulsions were evaluated for viscosity and appearance (micro/macroscopic) over 30 days, being evaluated at the initial time (D1), after 15 (D15), and 30 days (D30). Viscosity was measured on a DV-III Digital Rheometer (Brookfield, Stoughton, Massachusetts, USA) with a CP-40 spindle, analyzing 0.6 mL samples at 10.0–80.0 rpm. Macroscopic aspect was evaluated by visual analysis, seeking to identify a milky, opaque, low-viscosity emulsion, without phase separation. Photographic records were obtained. If separation occurred, the ratio between the thickness of the phases and the total thickness of the emulsion was calculated. The microscopic aspect was evaluated in a polarized light microscope (Zeiss Axio Imager M2, Carl Zeiss, Oberkochen, Germany) with an AxioCam digital camera (Model ERc 5S, Carl Zeiss, Oberkochen, Germany). Before analysis, a droplet of the diluted formulation (1:10 in mineral oil) was placed on a slide, covered. Images of the distribution of globules in the emulsions were obtained during the analyses. Data were processed with ZEN Lite 2012 software (Carl Zeiss Microscopy S.L., Germany).

**Supplementary Results**

**Fig. S1.** (A) Characterization of TT and PR8 antigens by SDS-PAGE. TT antigen titration curves using positive and negative sera (B) and PR8 with positive serum (C) and negative serum (D).

**Table S1.** Characteristics of the emulsions (1^st^ dose) loaded or not with the PR8 antigen, over 30 days. EMU-P: Emulsivant™ 70 emulsion without antigens; EMU-PR8: Emulsivant™ 70 emulsion with PR8; MONT-PR8: Montanide^TM^ ISA 70 emulsion with PR8; NM: no measurement (low amount of sediment); O: oil release ratio in the emulsion; S: proportion of sediment in the emulsion.

| Days (D) | D1 | | D15 (4°C) | | | D30 (4°C) | | |
| --- | --- | --- | --- | --- | --- | --- | --- | --- |
| Emulsions | **Viscosity (Cp)** | **Globule size (µm)** | **Viscosity (Cp)** | **Globule size (µm)** | **Phase separation ratio** | **Viscosity (Cp)** | **Globule size (µm)** | **Phase separation ratio** |
| EMU-P | 34.7 – 35.3 | 1.8 - 5.9 | 37.3 – 37.6 | 2.0 – 6.7 | 0.13 (O); 0.21 (S) | 34.7 – 35.3 | 1.4 – 5.8 | 0.16 (O); 0.32 (S) |
| EMU-PR8 | 35.6 – 36.0 | 1.8 – 5.4 | 36.0 - 36.3 | 1.6 - 6.6 | 0.14 (O); 0.22 (S) | 36.8 – 37.3 | 1.6 - 5.9 | 0.17 (O); 0.30 (S) |
| MONT- PR8 | 43.4 – 43.5 | 0.9 – 2.0 | 41.5 – 42.2 | 0.5 – 0.9 | 0.10 (O); NM (S) | 39.1 – 39.3 | 0.8– 1.5 | 0.26 (O); NM (S) |

**Fig. S2.** Production of IFN-γ in splenic cells of mice (vaccinated with different formulations) after ConA stimuli. EMU-P: Emulsivant™ 70 emulsion without antigens; EMU-TT: Emulsivant™ 70 emulsion with TT; MONT-TT: Montanide^TM^ ISA 70 emulsion with TT; PBS-TT: phosphate buffered saline with TT; C+: positive control (TT with Alhydrogel^®^ and CpG). Data presented as mean ± SEM. Different letters represent significant difference (*p* < 0.05; Kruskal-Wallis’s test with Dunn's post-test).

**Fig. S3.** Production of IFN-γ in splenic cells of mice (vaccinated with different formulations) after ConA stimuli, pre-challenge and post-challenge. EMU-P: Emulsivant™ 70 emulsion without antigens; EMU-PR8: Emulsivant™ 70 emulsion with PR8; MONT-PR8: Montanide^TM^ ISA 70 emulsion with PR8; PBS-PR8: phosphate buffered saline with PR8; C+: positive control (PR8 with Alhydrogel^®^ and CpG). Data presented as mean ± SEM. Different letters represent significant difference (*p* < 0.05; Kruskal-Wallis’s test with Dunn's post-test).

**Fig. S4.** Macroscopic characteristics of the emulsions (1^st^ dose) loaded or not with the PR8 antigen on day 15 (D15) and day 30 (D30) at 4ºC. The white arrow indicates the presence of sediment and were identified the oil release and sedimentation at the top and bottom of the bottles. No emulsion breakdown was observed for any of the formulations presented. EMU-P: Emulsivant™ 70 emulsion without antigens; EMU-PR8: Emulsivant™ 70 emulsion with PR8; MONT-PR8: Montanide^TM^ ISA 70 emulsion with PR8.
